# The phylogenetics of Anguillicolidae (Nematoda: Anguillicoloidea), swimbladder parasites of eels

**DOI:** 10.1186/1471-2148-12-60

**Published:** 2012-05-04

**Authors:** Dominik R Laetsch, Emanuel G Heitlinger, Horst Taraschewski, Steven A Nadler, Mark L Blaxter

**Affiliations:** 1 Department of Ecology and Parasitology, Zoological Institute 1, University of Karlsruhe, 76131, Karlsruhe, Germany; 2 Institute of Evolutionary Biology, The Ashworth Laboratories, The University of Edinburgh, EH9 3JT, Edinburgh, UK; 3 Department of Nematology, University of California, Davis, 95616, CA, USA

**Keywords:** Anguillicola, Anguillicoloides, Invasive, Host switch, Cryptic species, DNA-taxonomy, Barcoding

## Abstract

**Background:**

Anguillicolidae Yamaguti, 1935 is a family of parasitic nematode infecting fresh-water eels of the genus *Anguilla*, comprising five species in the genera *Anguillicola* and *Anguillicoloides*. *Anguillicoloides crassus* is of particular importance, as it has recently spread from its endemic range in the Eastern Pacific to Europe and North America, where it poses a significant threat to new, naïve hosts such as the economic important eel species *Anguilla anguilla* and *Anguilla rostrata*. The Anguillicolidae are therefore all potentially invasive taxa, but the relationships of the described species remain unclear. Anguillicolidae is part of Spirurina, a diverse clade made up of only animal parasites, but placement of the family within Spirurina is based on limited data.

**Results:**

We generated an extensive DNA sequence dataset from three loci (the 5' one-third of the nuclear small subunit ribosomal RNA, the D2-D3 region of the nuclear large subunit ribosomal RNA and the 5' half of the mitochondrial cytochrome *c* oxidase I gene) for the five species of Anguillicolidae and used this to investigate specific and generic boundaries within the family, and the relationship of Anguillicolidae to other spirurine nematodes. Neither nuclear nor mitochondrial sequences supported monophyly of *Anguillicoloides*. Genetic diversity within the African species *Anguillicoloides papernai* was suggestive of cryptic taxa, as was the finding of distinct lineages of *Anguillicoloides novaezelandiae* in New Zealand and Tasmania. Phylogenetic analysis of the Spirurina grouped the Anguillicolidae together with members of the Gnathostomatidae and Seuratidae.

**Conclusions:**

The Anguillicolidae is part of a complex radiation of parasitic nematodes of vertebrates with wide host diversity (chondrichthyes, teleosts, squamates and mammals), most closely related to other marine vertebrate parasites that also have complex life cycles. Molecular analyses do not support the recent division of Anguillicolidae into two genera. The described species may hide cryptic taxa, identified here by DNA taxonomy, and this DNA barcoding approach may assist in tracking species invasions. The propensity for host switching, and thus the potential for invasive behaviour, is found in *A. crassus*, *A. novaezelandiae* and *A. papernai*, and thus may be common to the group.

## Background

Anguillicolidae is a family of ichthyoparasitic nematodes endemic to fresh-water eels of the genus *Anguilla* around the Pacific and Indian Oceans. There are five morphospecies, recently divided into the genera *Anguillicola* (a single species, *Anguillicola globiceps*) and *Anguillicoloides* (four species: *Anguillicoloides crassus*, *Anguillicoloides papernai*, *Anguillicoloides australiensis*, and *Anguillicoloides novaezelandiae*; all were previously classified as *Anguillicola*) (see Table 1) [1-3]. Pre-adult and adult worms feed on blood from vessels within the wall of the swim bladder, and their basic life cycles, as far as known, are very similar, involving zooplanktonic and zoobenthic intermediate hosts (e.g. cyclopoids and ostracods) [4-8]. European populations of *A. crassus* have been described as being able to utilise several aquatic species (including freshwater teleosts, amphibians, gastropods and arthropods) as paratenic hosts [6,9-11]. In the last three decades, interest in this family of nematodes has been driven by *A. crassus* Kuwahara, Niimi and Itagaki 1974, which has spread from its native host, the Japanese eel *Anguilla japonica*, to parasitise European eels *Anguilla anguilla*, where it induces significant morbidity and mortality [12-15]. 

**Table 1:** Anguillicolidae sequences

**Species**	**nSSU**				**nLSU D2-D3**				**COX1**			
	**#**	**h**	**L**	**GC (%)**	**#**	**h**	**L**	**GC (%)**	**#**	**h**	**L**	**GC (%)**
*Anguillicoloides crassus*	56	1	867	46.94	54	2	653 663	51.43 52.22	49	29	550	30.55 - 31.64
*Anguillicoloides papernai*	33	1	867	47.06	35	4	651 663	51.89 52.53	22	13	550	30.55 - 34.91
*Anguillicoloides australiensis*	15	1	867	47.06	18	1	663	51.9	15	10	550	30.55 - 31.27
*Anguillicoloides novaezelandiae*	10	1	867	47.17	10	1	663	52.04	9	1	550	34.73
*Anguillicola globiceps*	6	1	867	47.47	6	1	651	51.31	1	1	550	32.73
Total	120	5	-	-	133	9	-	-	96	54	-	-

Data collected for three genes (nSSU 5': the 5' one third of the nuclear small subunit ribosomal RNA gene; nLSU D2-D3: the D2-D3 region of the nuclear large subunit ribosomal RNA gene; COX1: the 5' half of the mitochondrial cytochrome oxidase 1 gene) for each of the five species of Anguillicolidae. #: successfully sequenced fragments, h: the number of unique sequences found, L: the length (in bp) of the fragments, GC (%): the GC-content (in case of multiple sequences a range is given).

In East Asia, *A. crassus* parasitises the native Japanese *An. japonica* as well as introduced and cultured *An. anguilla* and *Anguilla rostrata* (the American eel) [16,17]. In its native host this parasite is minimally pathogenic, with a small adult body mass and low infection intensity [13]. In the 1980s the parasite was introduced from Taiwan into Europe as a result of the live eel trade [18,19], and the parasite colonised wild European eels, *An. anguilla*. In this new host the parasite attains much higher intensities. *Anguillicoloides crassus* infections have since spread through wild and farmed populations of *An. anguilla* in Europe and North Africa and are associated with cases of mass mortality when paired with environmental stressors such as high temperatures and low dissolved oxygen levels [20]. A two-stage colonisation pattern has been described, consisting of rapid spread upon introduction into a water system followed by equilibration at ceiling levels [21]. *A. crassus* was subsequently, and likely independently, introduced into populations of *An. rostrata* in North America. The introduction into North America is considered to have been from Japan [19,22-24]. *A. crassus* has also recently been reported from the island of Réunion near Madagascar, where it was found in three indigenous *Anguilla * species [25]. Being a “global invader” (*sensu *[26] and [13]), it is considered an important pathogen of the economically relevant Atlantic eel species *An. anguilla* and *An. rostrata *[15,27].

*A. novaezelandiae* Moravec and Taraschewski 1988 was first described from the short-fin eel *Anguilla australis* from New Zealand [2]. However, an explant population of this species was recorded in Lake Bracciano in Italy following stocking with *An. australis* in 1975 [28]. After the introduction of *A. crassus* into Lake Bracciano in 1993, the *A. novaezelandiae* population appears to have disappeared [29]. *A. australiensis* Johnston and Mawston 1940 is recorded only from North-East Australia where it parasitises the native long-fin eel *Anguilla reinhardtii *[2], and no evidence for pathological effects on the host have been found [30]. *A. papernai* Moravec and Taraschewski 1988 is endemic to South Africa and Madagascar where it parasitises the African long-fin eel *Anguilla mossambica*. *A. papernai* has been shown to be able to complete its life cycle in European intermediate and final hosts in laboratory infections [7]. *A. globiceps* Yamaguti 1935 is known from certain prefectures of Japan and provinces of China [31,32] where it infects wild populations of the Japanese eel *An. japonica*.

A phylogenetically correct and robust taxonomy of Nematoda is critical to understanding of biodiversity, biogeography and host-parasite coevolution [33]. In the case of the Anguillicolidae, it is important to understand the phylogenetic distribution of traits associated with colonisation of and pathogenicity in new hosts, such as the plasticity of life-cycle traits and the increased per-host intensity for invasive *A. crassus*. DNA sequence data are good characters for taxonomic inference (i.e. DNA taxonomy) and for analysis of the deeper phylogenetic history of organisms. However, loci with different rates of evolution are typically required to resolve these different types of questions.

The nuclear small subunit ribosomal RNA gene (nSSU or 18S) has been used extensively for analysis of nematode phylogenetics [34,35], but has limited resolution at the congeneric level, including within Anguillicolidae [36]. In analyses of Nematoda using nSSU, *A. crassus* is placed within the entirely animal parasitic Clade III (suborder Spirurina in order Rhabditida *sensu* De Ley and Blaxter [34,35,37,38]), consisting of the Gnathostomatomorpha, Ascaridomorpha, Oxyuridomorpha, Rhigonematomorpha and Spiruromorpha. De Ley and Blaxter [37,38] placed Anguillicolidae, along with other taxa in Dracunculoidea, as *incertae sedis* within Spirurina. Focussed analyses of Spirurina using nSSU have identified significant conflicts with both classical systematics and the revisions of De Ley and Blaxter [39,40], in particular the non-monophyly of 'Ascaridomorpha' and the placement of *A. crassus* together with the vertebrate-parasitic genus *Gnathostoma*, and not allied to other dracunculoids. The ichthyoparasitic cucullanid *Truttaedactinis truttae* was identified as sister to other analysed Spirurina [41]. Moravec in his synoptic revision [3] removed the *Anguillicolidae* from the superfamily Dracunculoidea and erected the superfamily Anguillicoloidea with the family Anguillicolidae as its only member. In addition, based on morphological characters, the sub-genera *Anguillicoloides* and *Anguillicola* were proposed to be promoted to the taxonomic rank of genera [3].

The nuclear large subunit ribosomal RNA gene (nLSU or 28S), in particular the section spanning diversity loops D1 and D3, is an attractive alternative to nSSU, as there are both highly conserved parts and regions of more rapid evolution. nLSU D1-D3 sequences have also been used for analyses of Spirurina [42], but the available data are much more sparse than for nSSU. A third candidate locus is the mitochondrial gene cytochrome oxidase I (COX1, specifically the 5' half or 'Folmer region'), which has been proposed as a universal DNA taxonomy and DNA barcoding target for Metazoa [43-47]. This mitochondrial locus evolves very rapidly compared to nSSU, such that there is appreciable variation within species. COX1 is beginning to be used widely in analyses of nematode population structure and phylogeography [48], including *Anguillicola/Anguillicoloides *[19], generating extensive datasets with, however, low taxonomic coverage.

The assumption underpinning DNA taxonomy is the existence of a “barcoding gap”, resulting from the non-overlapping, discrete distribution of intra- and inter-specific variation of the DNA fragment analysed [49]. Given this, unidentified specimens that differ by less than a threshold sequence divergence from reference voucher sequences (from specimens reliably identified to species, for example) can be assigned to that species. Symmetrically, specimens yielding sequences that are more divergent than the specified threshold can be allocated to a different taxon. For Metazoa, a general COX1 DNA taxonomy threshold of 2% has been proposed [50], but molecular operational taxonomic units (MOTU) [44] can be defined at any cutoff, and exploration of the pattern of MOTU count and cutoff is warranted for previously understudied groups [51]. This approach can simplify the identification of potentially morphologically cryptic taxa and encourage revisionary taxonomy [52].

To investigate boundaries of species and genera in Anguillicolidae, and assess placement of this family in the diversity of Spirurina, we have generated data from nSSU, nLSU and COX1 for a large sample of Anguillicolidae encompassing all five nominal species. We assessed the empirical support for barcoding gaps in the three genes by analysing the impact of increasing sequence divergence thresholds, and the congruence between MOTU derived from different loci and species assignments. We analyse and compare the ability of the three genes to resolve the evolutionary relationships between the Anguillicolidae and test whether the morphological taxonomy of this family is supported by molecular evidence. Additionally, we infer a phylogenetic tree for Spirurina based on published and new nSSU sequences, to further refine our understanding of the relationships of the Anguillicolidae, and thus better inform hypotheses as to their origins.

## Results

### Multi-locus marker development from Anguillicolidae

One hundred and fifty anguillicolid specimens were obtained representing all five morphologically defined species [2]. Primer sets were verified, and three genes (nSSU, nLSU and COX1) were amplified and sequenced (Table 1 summarises these data; locality data for specimens are listed in Table 2). We also amplified and sequenced these genes from an unidentified nematode larva (SNR118) recovered from the serosa of the swim bladder of *An. mossambica* from South Africa. Not all specimens yielded amplicons for all genes. For 79 specimens we obtained good sequences for all three genes and these form the nSSU*, nLSU* and COX1* datasets analysed below. For MOTU analyses we also considered an augmented COX1 dataset comprising all the sequences generated in this project and also additional published sequences from *A. crassus* and *A. novaezelandiae *[19] for a total of 452 sequences. 

**Table 2:** Location of sampling sites

**Prefix**	**Site (Country)**	**Geodetic coordinates**		**Host species**	Collector* and date**
		**Latitude**	**Longitude**		
AQT	Townsville, Queensland (Australia)	19°18'S	146°44'E	*An. reinhardtii*	BS 2007
AQB	Brisbane, Queensland (Australia)	27°38'S	153°12'E	*An. reinhardtii*	BS 2007
ATD	Deloraine, Tasmania (Australia)	41°31'S	146°39'E	*An. australis*	LP 2008
CGG	Guangzhou, Guangdong (China)	23°07'N	113°15'E	*An. anguilla*	HT 03/07
CGZ	Zhuhai, Guangdong (China)	22°16'N	113°34'E	*An. anguilla*	HT 03/07
EAV	Albufera de Valencia (Spain)	39°21'N	0°20'W	*An. anguilla*	PMR 01/09
GST	Steinfeld (Germany)	49°02'N	8°02'E	*An. anguilla*	AK 2009
GRA	Rußheimer Altrhein (Germany)	49°12'N	8°25'E	*An. anguilla*	EH, UW 2009
JPN	Natural water system, Wakayama (Japan)	34°13'N	135°10'E	*An. japonica*	HS 2006 2007
MAD	Ambatondrazaka (Madagascar) ***	17°83'S	48°41'E	*An. mossambica*	OW 05/2008
POL	Sniardwy Lake, Mikolajki (Poland)	53°45'N	21°43'E	*An. anguilla*	UW 2009
POR	Ribeira das Lampreias (Portugal)	38°47'N	9°01'W	*An. anguilla*	JLC 03/09
SFH	Farm Dam, Fort Hare (South Africa)	32°47'S	26°50'E	*An. mossambica*	HT 03/08
SKR	Koonap River (South Africa)	32°1'S	26°08'E	*An. mossambica*	HT 03/08
SSD	Sunday’s River, Slagboom Dam (South Africa)	33°22'S	25°40'E	*An. mossambica*	HT 03/08
SDD	Sunday’s River, Darlington Dam (South Africa)	33°12'S	25°8'E	*An. mossambica*	HT 03/08
SGF	Great Fish River (South Africa)	33°05'S	26°46'E	*An. mossambica*	HT 03/08
SNR	Nahoon River (South Africa)	32°54'S	27°48'E	*An. mossambica*	HT 03/08
TUR	Asi River, Hatay (Turkey)	36°24'N	36°21'E	*An. anguilla*	EG 12/08
TKR	Sinyuan, Kaoping River (Taiwan)	22°30'N	120°25'E	*An. japonica*	HT 9/06
TCU	Eel culturing pond, Budai, (Taiwan)	22°38'N	120°26'E	*An. japonica*	HT 9/06

Sampling locations for the nematodes including label prefix, information on geographic position (latitude and longitude), host species and information about sampling.

* Collectors are identified by their initials: AK: Albert Keim, BS: Björn Schäffner, EH: Emanuel Heitlinger, EG: Ercüment Genç, HT: Horst Taraschewski, JLC: José Lino Costa, LP: Lea Perseke, OW: Olaf Weyl, PMR: Pilar Muñoz Ruíz, KG: Kerstin Geiss and HS: Hiroshi Sato.

** Month (where known) and year(s).

*** *A. papernai* were obtained from *An. mossambica* that were purchased from a commercial eel supplier. According to the supplier, all eels originate from tributaries of the Mangory River. The exact localities of capture, holding conditions and periods prior to collection are however unknown.

### Species boundaries and MOTU in Anguillicolidae

**Figure 1:**
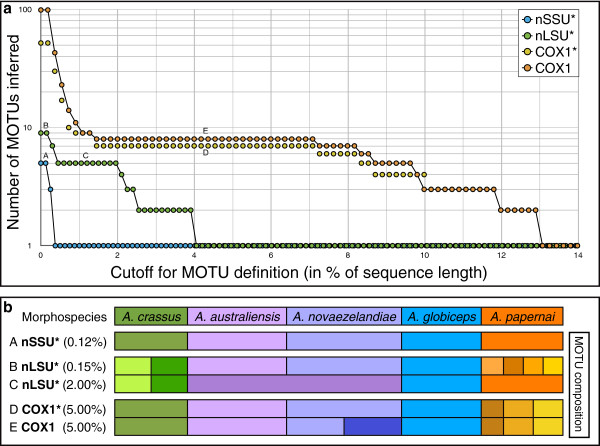
**MOTU analysis of three marker loci from Anguillicolidaea.** Variation in the number of MOTUs inferred at cut-offs ranging from 0 - 14% sequence divergence for specimens for which all three genes were sequenced (nSSU*, nLSU*, COX1*). Results of the MOTU analysis of the expanded COX1 dataset are included for comparison. Critical cutoff intervals for the different datasets are indicated in letters (A – E). **b.** Comparison of morphological species identified *sensu* Moravec and Taraschewski [2] with MOTU composition at the critical cutoff intervals (A – E).

We used jMOTU [51] to infer the numbers of MOTU for each gene across a wide range of cutoffs (Figure 1). There were five unique sequences in the nSSU* dataset, and five MOTUs between 0 – 0.12% sequence divergence (equivalent to a 0 – 1 bp cutoff value) and these MOTUs were consistent with the morphological identifications of the specimens. At 0.25% sequence divergence cutoff three MOTUs were defined (all *A. globiceps*; all *A. crassus*; combined *A. papernai*, *A. australiensis*, *A. novaezelandiae*). At 0.37% divergence, all the specimens were grouped into a single MOTU. There were nine unique sequences in the nLSU* dataset, and for up to 0.15% sequence divergence, independent MOTUs were defined for *A. australiensis*, *A. novaezelandiae* and *A. globiceps*. However, at this cutoff there were four MOTUs for *A. papernai* and two for *A. crassus*. An apparent barcoding gap (plateau in MOTU number) is evident at cutoffs between 0.5 – 2.0% sequence divergence, yielding 5 MOTUs. These MOTUs do not correspond to the morphological species, as one groups all sequences from *A. australiensis* and *A. novaezelandiae*, while *A. crassus* sequences are divided into two MOTUs. Within the COX1* dataset, 52 discrete sequences were found. There is a clear barcoding gap yielding seven MOTUs from 1.5 – 7.1% sequence divergence. In this span MOTU composition resembles morphological species boundaries, except that *A. papernai* sequences are divided into 3 MOTUs. Five MOTUs are observed only for the narrow range of 8.3 – 8.5% sequence divergence, corresponding to *A. crassusA. globiceps*, two MOTUs identified as *A. papernai* and one MOTU combining *A. australiensis* and *A. novaezelandiae*. The complete COX1 dataset had a total of 99 different sequences, but these had little impact on MOTU composition above a cut-off of 1.5% sequence divergence. However, MOTU richness was increased by one across the potential barcoding gap, due to sequences from specimens identified as *A. novaezelandiae* from New Zealand forming a distinct MOTU from other *A. novaezelandiae* from Tasmania. 

### Phylogenetic analyses of Spirurina

**Figure 2:**
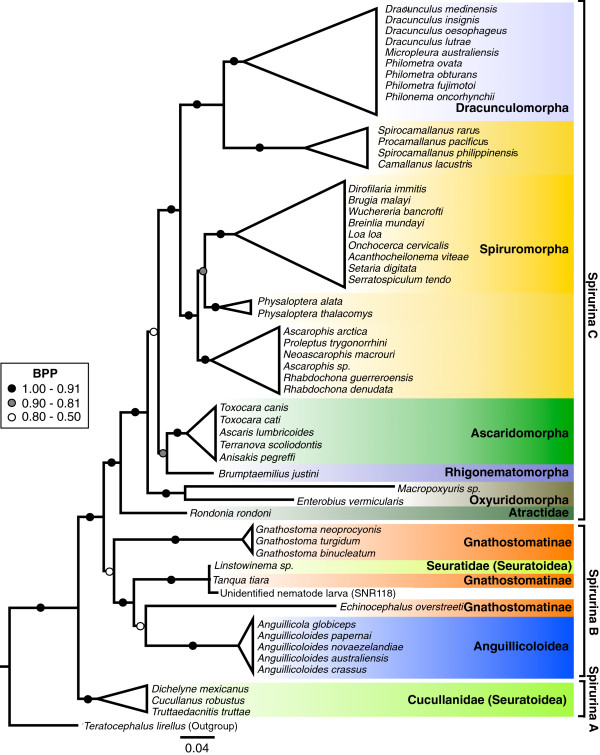
**Phylogenetic analysis of nSSU sequences of Spirurina.** Consensus phylogram of the analysis of the nSSU sequences from Spirurina using Bayesian Inference, rooted with *Teratocephalus lirellus*, a non-spirurine rhabditid. Branches are collapsed where possible based on taxonomic affiliations and major groups are highlighted. Bayesian posterior probabilities for internal branches are indicated. The scale bar indicates the average expected number of substitutions per site.

We used these new sequences in conjunction with existing data to investigate the placement of Anguillicolidae within the Spirurina, and the interrelationships of Anguillicolidae species. The overall branching order of the phylogenetic tree of the Spirurina, inferred using nSSU sequences (Figure 2) was consistent with results of previous analyses [39-41]. Spirurina can be divided into three subclades. Spirurina "A", encompassing the cucullanid nematodes *Dichelyne mexicanus*, *Cucullanus robustus* and *Truttaedacnitis truttae* (Seuratoidea), is the sister group to the other Spirurina. Spirurina "B" comprises members of the Gnathostomatidae, Seuratidae and Anguillicolidae, as well as the unidentified eel-parasitic larva SNR118, whereas the sister group, Spirurina "C", consists of the remaining taxa, including Oxyuridomorpha, Spiruromorpha, Ascaridomorpha, Rhigonematomorpha and the remaining Dracunculomorpha. Neither Gnathostomatinae Railliet 1985 (represented by the genera *Echinocephalus*,*Tanqua* and *Gnathostoma*) nor Seuratoidea Chabaud Campan-Rouget et Brigoo, 1959 are monophyletic, as most recent common ancestors are shared between *Linstowinema* sp. (Seuratoidea) and *T. tiara* (Gnathostomatinae), and between Anguillicolidae and *E. overstreeti* (Gnathostomatinae). 

### Phylogenetic relationships within the Anguillicolidae

**Figure 3:**
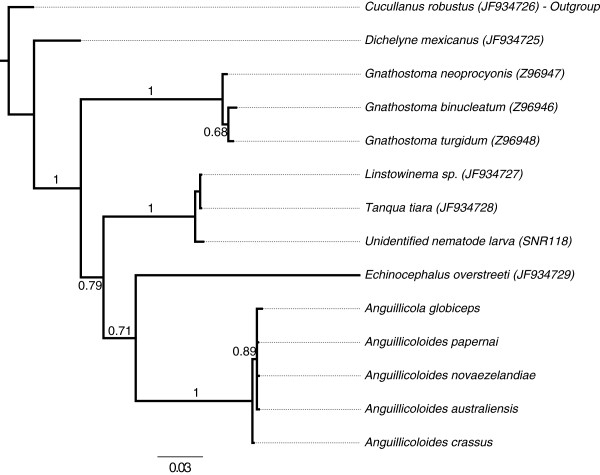
**Phylogenetic analysis of nSSU sequences of Spirurina B.** Consensus phylogram of the analysis of the nSSU sequences from Spirurina B using Bayesian Inference, rooted with *Cucullanus robustus* (Spirurina A). Bayesian posterior probabilities for internal branches are indicated. The scale bar indicates the average expected number of substitutions per site.

**Figure 4:**
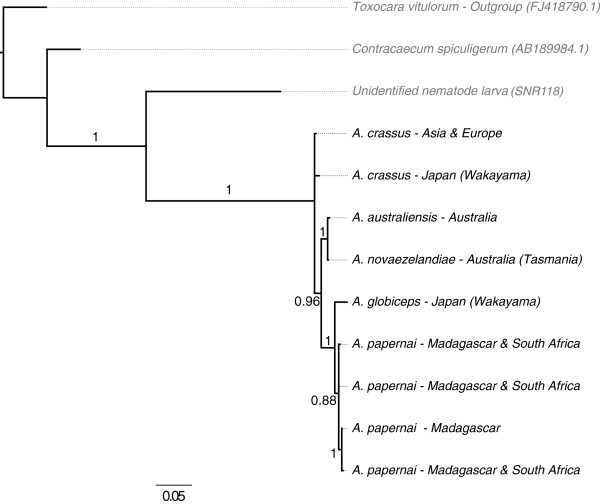
**Phylogenetic analysis of nLSU sequences of Anguillicolidae.** Consensus phylogram of the analysis of the nLSU sequences from Anguillicolidae and outgroups using Bayesian Inference. Bayesian posterior probabilities for internal branches are indicated. The scale bar indicates the inferred number of base substitutions per site.

**Figure 5:**
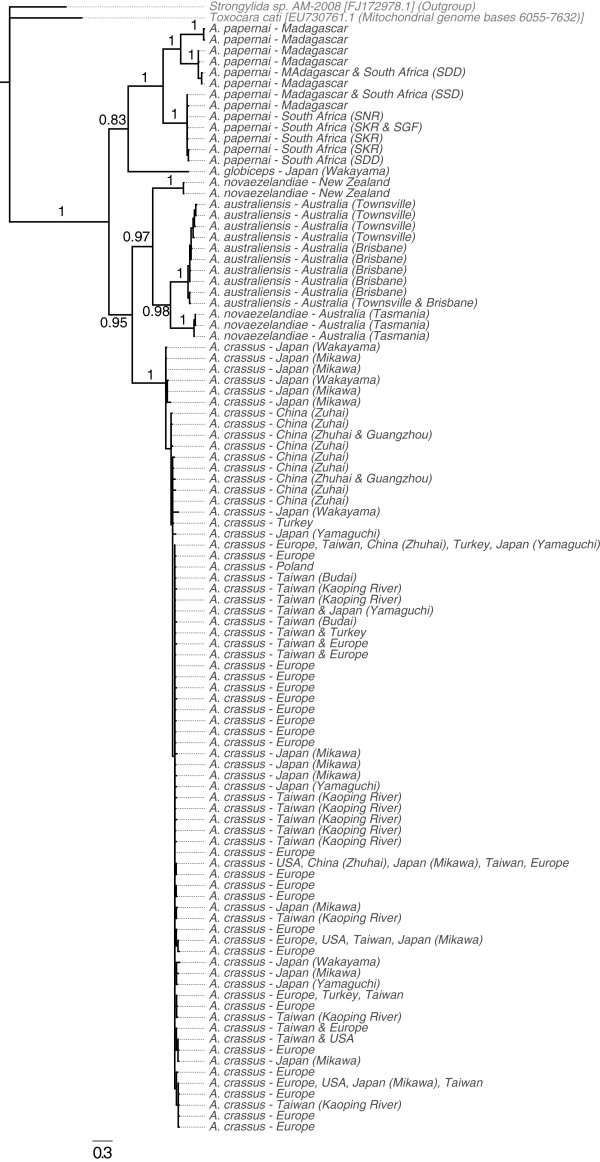
**Phylogenetic analysis of COX1 sequences of Anguillicolidae.** Consensus phylogram of the analysis of the COX1 sequences from Anguillicolidae and outgroups using Bayesian Inference. Bayesian posterior probabilities for internal branches are indicated. The scale bar indicates the average expected number of substitutions per site.

Both nuclear (Figures 2, 3, and 4) and mitochondrial (Figure 5) loci supported the monophyly of the Anguillicolidae (the Bayesian posterior probability [BPP] of monophyly is 1.0 in all analyses). Phylogenetic analyses of both nuclear loci, nSSU (Figures 2 and 3) and nLSU (Figure 4), placed *A. crassus* as sister to the other four anguillicolids. We note that the resolution of these analyses is likely limited by the low numbers of parsimony informative sites (PIS) present in either region (nSSU_PIS_ = 5, nLSU_PIS_ = 42).

Analysis of COX1 (Figure 5) divided the Anguillicolidae into two lineages with respect to morphospecies: a weakly supported clade formed by *A. globiceps* and *A. papernai* (with BPP of only 0.83 for COX1, but 1.00 for nLSU), and a robustly supported clade consisting of *A. crassus*, *A. novaezelandiae* and *A. australiensis*. A close relationship of the Oceania species *A. novaezelandiae* and *A. australiensis* was strongly supported (BPP for nLSU = 1.00 and for COX1 = 0.97). In the COX1 analyses, the *A. novaezelandiae* population sampled in Tasmania is more closely related to *A. australiensis* than to the *A. novaezelandiae* population from New Zealand. Mirroring the MOTU analyses, there is significant divergence within *A. papernai*, with three strongly supported clades (COX1 BPP = 1.0) as distinct from each other as are Tasmanian *A. novaezelandiae* from *A. australensis*.

### Population diversity in *Anguillicola crassus*

**Figure 6:**
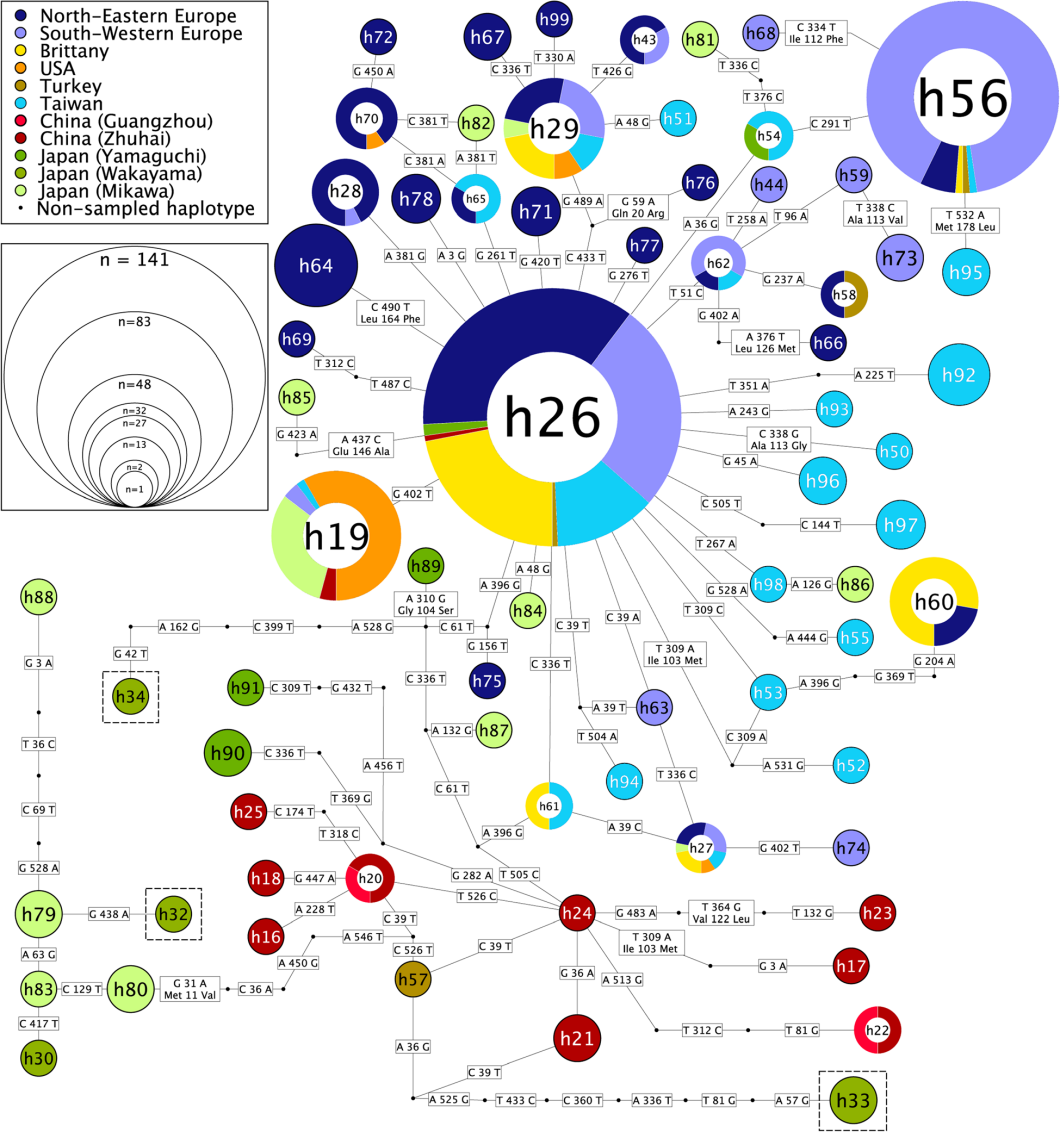
**Network analysis of COX1 sequences from *Anguillicola crassus.*** Statistical parsimony network of 70 distinct *A. crassus* COX1 sequences. The three COX1 haplotypes containing four specimens that have the minority *A. crassus* 28 S rDNA D2-D3 haplotype are highlighted. A list of all specimens associated with each COX1 haplotype can be found in the Additional file 1.

Previous analyses of COX1 and microsatellite diversity in *A. crassus *[19] have defined the likely origin of the European populations (by transfer from Taiwan) and the effects of isolation-by-distance on the structure of these European populations. Analysis of our COX1 data in conjunction with these previous data reinforces patterns observed previously [19] (Figure 6), with East Asian populations harbouring the greatest diversity, European and North American populations being closely related to distinct East Asian populations, and European populations showing some stratification by geographical location. However, in our study additional unique haplotypes were found from Turkish sampling locations that were grouped with haplotypes from Taiwan and China. 

## Discussion

### Anguillicolidae species diversity analysed using DNA taxonomy

Primers that selectively amplify DNA from certain taxa are crucial to surveys in which parasite DNA has to be amplified over the background of host DNA. Studies on Anguillicolidae clearly fall into this category due to the haematophagous life-style of the pre-adult (larval) stages, and thus host blood cells in their digestive system. The primers used here [34,53,54] were broadly successful in amplification of the desired nematode fragments for subsequent sequencing. Thus DNA barcoding approaches are readily applicable to these nematodes.

Although there were five distinct nSSU sequences, corresponding to the five morphological species, these sequences differed by no more than 3 bases over the 867 bases sequenced. This close similarity raises the issue of potential misidentification due to sequencing errors, and thus nSSU may not be useful for species-level identification surveys of Anguillicolids. As expected the nLSU D2-D3 region displayed higher intra- and inter-specific variation among morphospecies than nSSU, with 9 unique sequences. This variation, along with the presence of indels, should also enable the design of species-specific amplification primers. This nLSU fragment has been successfully amplified from partially degraded specimens of *A. crassus* encapsulated within host tissue [55], and from morphologically indistinguishable life stages such as larvae. nLSU could be used for PCR-based diagnostics of anguillicoloidosis in eels and can serve as a reliable tool in life-cycle studies.

The methodology of MOTU definition, as implemented in jMOTU [51], explores a defined range of sequence divergence thresholds upon which a plateau of MOTU richness, i.e. potential barcoding gaps, can be identified. For both the nuclear loci studied, no clear evidence of a barcoding gap was found. The lack of interspecific diversity for the nSSU locus limits the utility of this marker at congeneric levels. Multiple within-(morpho)species nLSU sequence types were only observed in *A. crassus* (two sequence types) and *A. papernai* (four sequence types). One *A. crassus* sequence type is found globally, while the second is confined to one Japanese population, supporting the hypothesis of an Asian origin of this species [19].

The highest number of sequence types (54) was observed for the COX1 fragment, which has previously been used to investigate global population structure in *A. crassus *[19]. We were able to obtain only a single COX1 fragment from *A. globiceps* that contained a correct open reading frame, despite multiple trials. This phenomenon has been observed in other surveys of this species (S. Wielgoss, pers. comm.), and may be due to preferential amplification of nuclear copies of mitochondrial DNA [48] or the existence of RNA editing of this mitochondrial transcript, as has been observed in other nematodes [56]. Sequencing of COX1 transcripts and the mitochondrial genome may be informative.

Our *A. crassus* COX1 data, when added to those previously determined, reinforces the view that the invasion of West European and North American hosts has only happened a few times (likely once for each location) and that the origins of the invading parasite differ. North American *A. crassus* are robustly linked to Japanese specimens, and European to Taiwanese. By sampling additional locations across China and Japan, we identified many additional, unique COX1 haplotypes limited to East Asia, affirming this as the area of highest diversity, and thus the likely origin of diversity of this species. Interestingly, Turkish *A. crassus* may have distinct origins, as some haplotypes group with Chinese and Taiwanese sequences not previously observed in Western Europe.

The COX1 locus of the Anguillicolidae exhibits a likely barcoding gap with over 5.6% sequence divergence, exceeding by more than twofold the proposed threshold (2%) for Metazoa [57]. Although the species *A. crassusA. globiceps* and *A. australiensis* were stably grouped into individual MOTUs within this plateau, sequences of both *A. novaezelandiae* and *A. papernai* were found in multiple, distinct COX1 MOTUs. These distinct MOTUs within a single nominal species are possible evidence of cryptic speciation. The Madagascar populations of *A. papernai* may be the source of introduction into South Africa, since all sequence types from the five sampling sites in South Africa occur in, or are closely related to, those from Madagascar. This inference is supported by the pattern observed for the four nLSU sequence types of *A. papernai*, which are all present in Madagascar. We note that the mitochondrial locus COX1 has uniparental inheritance and thus may not be an unbiased reporter of phylogeographic history, but propose these hypotheses as testable inferences from our data.

Specimen SNR118 was a single, unidentified larva from the swim bladder serosa of a South African *A. mossambica*, and the whole specimen was used for DNA extraction. Its nSSU sequence showed that it is a member of Spirurina B. Sampling site, host species and phylogenetic position in the Spirurina suggest this specimen belongs to or is closely affiliated to *Paraquimperia africana* (Seuratoidea) [58].

### DNA taxonomy and molecular phylogenetic systematics of the Anguillicolidae

We found no support for the division of the Anguillicolidae into the two genera *Anguillicola* and *Anguillicoloides* as proposed by Moravec [3], as *Anguillicola globiceps* was recovered within the radiation of *Anguillicoloides* species, suggesting that that the morphological criteria used to erect these putative genera (spinosity of cuticular ornamentation and the structure of the oesophagus) may not be phylogenetically informative. As noted previously [59], modifications of the structure of the oesophagus, a trait related to the mode of nutrition, may occur independently in the course of trophic adaptations. Our data support a single generic division for the species in Anguillicolidae, which by priority should be called *Anguillicola*, restoring *Anguillicoloides crassus* to *Anguillicola crassus* and ensuring continuity with historical literature on this important species.

Based on the analysis of the DNA sequences of three genes, a reevaluation of the taxonomy of the Anguillicolidea may be necessary. We identified eight discrete COX1 MOTUs over a wide range of divergence, up to 5%, suggesting that there may be eight species-level taxa represented if a barcoding cutoff of 2% is accepted. Three MOTUs are congruent with morphological species identifications: *A. crassus*, *A. globiceps* and *A. australiensis*. However specimens unambiguously assigned to the morphological species *A. novaezelandiae* and *A. papernai* are found in multiple MOTUs (two MOTUs for *A. novaezelandiae* and three for *A. papernai*). These additional MOTUs could represent morphologically cryptic, or previously unrecognised species.

In the case of *A. novaezelandiae* phylogenetic analyses of the COX1 locus suggest the paraphyly of this species, as the specimens from New Zealand (that form one MOTU) are robustly placed as the sister group to the clade consisting of *A. novaezelandiae* specimens from Tasmania (the second MOTU) plus *A. australiensis*. Since nLSU D2-D3 sequences are not available for *A. novaezelandiae* from New Zealand, additional representatives should be sampled to more thoroughly test the monophyly of this species.

*A. papernai* displays the greatest number of nLSU D2-D3 sequence types among the Anguillicolidae and its COX1 locus displays a clear division into three clades and is represented by three MOTUs. Whether these are distinct species level taxa, or merely distinct diverse populations of a widespread species will require analysis of additional loci. *A. papernai* is found in well separated watersheds, with no linking waterways, and an absence of human-induced admixture through stocking. The diversity within this species, together with the observation that it is able to complete its life cycle in European eel hosts [7], supports the need for further research.

A similar branching pattern is observed for both nLSU and COX1 for all species analysed except *A. crassus*. Analyses of the two nuclear loci revealed *A. crassus* to be sister to the other sampled species within Anguillicolidae. However the mitochondrial COX1 gene placed it as the sister taxon to the clade comprising *A. australiensis* and *A. novaezelandiae*. Despite the low number of informative sites in the nLSU and nSSU loci, we favour the hypothesis based on the nuclear loci, as no close outgroup sequences were available for COX1 and the sequences used (*Toxocara cati* and *Strongylida sp.*) may have impacted on proper rooting of the COX1 tree. Reanalyses with COX1 data from Seuratoidea or Gnathostomatinae would address this issue.

These data suggest a scenario of Asian origin for the Anguillicolidae, since *A. papernai* is the only endemic species west of the 80 degree line of longitude. Speciation of *A. crassus* could have taken place in East Asian waters, followed by the cladogeneses of the Oceanian species and the clade comprising *A. papernai* and *A. globiceps*. The biogeographic distribution of *A. papernai* observed today could thus be explained by a host-related dispersal of its ancestor.

**Figure 7:**
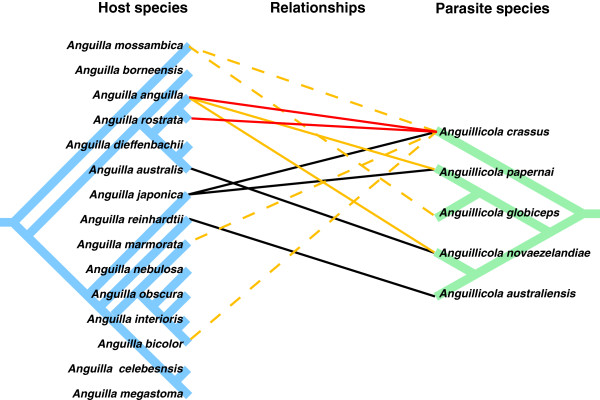
**Host switching in the Anguillicolidae.** Cladograms of the eel genus *Anguilla* (left tree, blue) [60] and its swim bladder parasites, the family *Anguillicolidae* (Nematoda: Anguillicoloidea) (right tree, green). The known host parasite relationships are indicated by: black lines = traditional host-parasite relationship, displaying low abundances and low pathogenicity (parasite endemic in host); red lines = novel host-parasite relationship, displaying high abundances and pathogenicity; orange lines = novel host-parasite relationship, displaying low abundances and pathogenicity; dashed orange lines = novel host-parasite relationship where completion of the life cycle has not been demonstrated.

Comparing the Anguillicolidae phylogenies with those of their hosts [60] we can infer several host switches within the nematodes, even excluding recent host-range expansion by *A. crassus* and *A. novaezelandiae* (Figure 7). The host phylogeny includes a deep split between an Indo-Pacific group, including *An. japonica*, and an Oceania/Atlantic group, including *An. anguilla* and *An. australis*. *A. australiensis*, parasitising *An. reinhardtii* in the Indo-Pacific host group is sister to *A. novaezelandiae* parasitising *An. australis* in the Oceania host group. If the relationship between Anguillicola and its eel hosts was established in an ancestral Indo-pacific host, a minimum of three host-capture events, two of them across the major split in the eel phylogeny, are required to explain the endemic distribution of these eel parasites. The recent proven ability of *A. crassus* to exploit new, phylogenetically well-separated hosts may be a reflection of this evolutionary propensity for switching. 

### Phylogenetic relationships within Spirurina

Spirurina includes parasites with a direct lifecyle as well as those that include biological vector and transport (paratenic) hosts in complex, multi-species systems [61]. Although some applications of parsimony principles might suggest that the simpler, direct life cycle should be ancestral for the group, molecular phylogenetic analyses robustly place two clades of parasites (Spirurina A and B) that have complex life histories as successive sister groups, at the base of the Spirurina, to clades that include direct life cycle parasites. This phylogenetic hypothesis favors intepretation of complex life histories as the ancestral state for the group. We found that members of the ichthyoparasitic family Cucullanidae (Seuratoidea: C. *robustusD. mexicanus* and *T. truttae*) form a well-supported clade (Spirurina A; Figure 3) that is sister to the remaining Spirurina [40,41]. Anguillicolidae are placed in Spirurina B along with certain members of the superfamily Seuratoidea and all sampled Gnathostomatoidea. Spirurina C includes the remaining taxa including the abundantly sampled Ascaridomorpha and Spiruromorpha. Previous analyses of taxa in Spirurina C have suggested that many classical groups are non-monophyletic, in particular "Ascaridomorpha" and "Spiruromorpha" [39,40,42], a result echoed here. The position of the three *Gnathostoma* species (*G. binucleatumG. neoprocyonis* and *G. turgidum*) outside of Spirurina C has been observed previously [40] and was recovered consistently in all analyses. Members of the other two genera of the Gnathostomatinae, i.e. *T. tiara* and *E. overstreeti*, are also displaced, and share direct ancestors with *Linstowinema* sp. (Seuratoidae) and members of Anguillicolidae, respectively. This result indicates the paraphyly of both Gnathostomatinae and Seuratoidea. The Dracunculoidea is also rendered paraphyletic, as members are found in Spirurina B and Spirurina C. This phylogenetic hypothesis reveals an enormous definitive host diversity within the Spirurina B, comprising fresh-water teleosts (Anguillicolidae), chondrichthyes (*Echinocephalus*), mammals (*Gnathostoma* and *Linstowinema*) and squamates (*Tanqua*), and an even wider diversity of vector and paratenic hosts. Denser taxon sampling in the Spirurina, with special emphasis on the morphologically diverse Spirurina B and C, is required to fully explore this fascinating group.

## Conclusions

We have investigated the genetic diversity within, and genetic distinctiveness of the five described species of the family Anguillicolidae. We found no support for the erection of two genera within the family, and identified two species (*A. novaezelandiae* and *A. papernai*) where within-species divergences and phylogenetic tree topology suggests the presence of distinct cryptic taxa. The role of host vicariance in the speciation of *A. globiceps* and *A. papernai* is an intriguing topic for future study, as is the evolutionary history of the Oceanian species (*A. australiensis* and the two MOTUs within *A. novaezelandiae*). We revisited the phylogeny of the Spirurina and identified a clade of vertebrate-parasitic taxa that includes Anguillicoloidae and members of other families, which must therefore be suspected of being paraphyletic. Our analyses highlight the possibility that *A. papernai* might transfer to and be pathogenic in new, economically important, eel hosts, and the data generated here will, we hope, act as reference for future DNA barcoding surveys of eel swim bladder parasites worldwide.

## Methods

### DNA extraction and sequencing

Sampling and identification of nematode specimens was performed by José Lino Costa, Kerstin Geiss, Ercüment Genç, Emanuel Heitlinger, Albert Keim, Lea Perseke, Pilar Muñoz Ruíz, Hiroshi Sato, Björn Schäffner, Horst Taraschewski, Urszula Weclawski and Olaf Weyl. A total of 150 anguillicolid nematodes were extracted from the swim bladders of their respective hosts from 21 different locations (see Table 2). In addition, an unidentified nematode larva (SNR118) was extracted from the serosa of the swim bladder of a specimen of *A. mossambica* from South Africa. DNA was prepared from single nematodes as described in [48]. Lysates were used directly as templates in PCR reactions. For all loci, 25 μl polymerase chain reactions (PCRs) were carried out using 0.1 μl Taq DNA Polymerase (5 units/μl) (Quiagen, Hilden, Germany), 2.7 μl 10x PCR Buffer (containing 15 mM MgCl_2_) (Quiagen), 2.7 μl 2 mM dNTP (2 mM dATP, dTTP, dGTP, dCTP), 0.4 μl of each PCR primer (10 μM), 2 μl template DNA and 17.1 μl ddH_2_O (Milli-Q). For partial nSSU amplification the forward primer SSU_F04 5’-GCTTGTCTCAAAGATTAAGCC-3’ and the reverse primer SSU_R26 5’-CATTCTTGGCAAATGCTTTCG-3’ [34] were used. Amplification of the nLSU D2-D3 region was carried out using forward primer D2A 5’-ACAAGTACCGTGAGGGAAAGT-3’ and the reverse primer D3B 5’-TGCGAAGGAACCAGCTACTA-3’ [53]. The cytochrome *c* oxidase subunit I (COX1) was amplified using the forward primer LCO1490 5’-GGTCAACAAATCATAAAGATATTGG-3’ and the reverse primer HCO2198 5’-TAAACTTCAGGGTGACCAAAAAAT-3’ [54]. PCR products were purified using shrimp alkaline phosphatase and *Escherichia coli* exonuclease I (USB Corporation, USA) as described in [62] and sequenced on an automated ABI Prism 3730 Genetic Analyzer using ABI BigDye v3.1 Terminator sequencing chemistry (Applied Biosystems, Foster City, CA) in the GenePool Genomics Facility, Edinburgh ( http://genepool.bio.ed.ac.uk). Sequencing of each PCR product was carried out in both directions to minimise PCR artefacts, ambiguities and base-calling errors. Directly sequenced COX1 PCR products of *A. globiceps* displaying indels disrupting the open reading frame were cloned (PCRII Topo TA cloning kit, Invitrogen) and only specimens for which a single, correct open reading frame (ORF) bearing sequence was identified have been included in the analyses. Raw ABI chromatograph files of the sequences were processed using trace2seq.pl (a perl program that uses phred [63,64] to identify high-quality base calls; A. Anthony and M. Blaxter, unpublished). After screening for contaminants using NCBI BLAST [65], the high quality forward and reverse sequences of each gene from each sample were aligned and a consensus sequence was inferred using a phred score of 30, i.e. a 99.9% accuracy of a base-call, as the detection threshold, resulting in an increase in sequence lengths and an improvement in sequence reliability. The 352 sequences were deposited in the EMBL database (accession numbers JF805371 – JF805722). Aligned sequence datasets for all analyses are available as Additional file 1 and also at datadryad.org under doi:10.5061/dryad.8h5p7p00.

### Construction of the datasets

For each of the three genes, an individual dataset was created (nSSU, nLSU and COX1) containing the respective consensus sequences from different individual nematode specimens. Trimmed datasets containing only sequence types derived from the subset of 79 specimens for which all three genes were successfully sequenced (termed nSSU*, nLSU* and COX1*). Additional COX1 sequences [19], obtained using the same primers, were retrieved from GenBank and included in the COX1 dataset. Sequences contained in the nSSU and COX1 datasets were of uniform length (787 bp and 550 bp, respectively) and could be aligned unambiguously using CLUSTALW 2.0.9 [66] with the penalties for gap opening and extension set to 10 and 0.2, respectively. Sequences in the nLSU alignment showed several insertion/deletion events (indels), which were binary coded for phylogenetic analyses [67] implemented in Seqstate 1.4 [68]. The data was included in the phylogenetic analysis as a binary partition under the restriction site model with the ascertainment bias set to variable, as suggested by [69].

### MOTU definition

jMOTU 1.0.7 [51] is a Java application that defines molecular operational taxonomic units (MOTUs) based on nucleotide sequences and a user defined range of cutoff values, the maximum number of base pair differences between two sequences, by using global alignment. MOTUs were defined at cut-off values ranging from 0 to 14% sequence divergence in intervals of 1 base pair, on sequences from specimens for which all three genes were sequenced (nSSU*, nLSU* and COX1*). To investigate the degree of influence sampling depth has on MOTU richness and membership, the results were compared to those of the MOTU analysis performed on the COX1 dataset.

### Phylogenetic analysis

For phylogenetic analysis of the Spirurina, additional sequences were retrieved from GenBank [39-41] and aligned to the anguillicolid sequences. New sequences from spirurine nematodes from a dataset provided by S. Nadler were also included [accession numbers JF934725–JF934737]. Regions within the resulting Spirurina alignment in which determination of homology was ambiguous (i.e. many apparently independent insertion-deletion events over 10 contiguous bases of the alignment) were excluded. For both the nLSU and COX1 data, outgroups were chosen based on closest sequence matches in the public databases, while for nSSU outgroups were chosen based on previous analyses. Bayesian phylogenetic inference was carried out using MrBayes 3.1.2 [69]. Phylogenies for individual datasets were inferred under the GTR + I + Γ model of sequence evolution, partitioned by codon position for COX1 and partitioned by datatype for nLSU (i.e. nucleotide data and binary insertion-deletion data). For each analysis, two independent Markov chain Monte Carlo (MCMC) runs of four Metropolis-coupled chains were performed with the gamma shape parameter, the proportion of invariable sites, base frequencies and substitution rates unlinked across partitions and assuming default priors. Chains were sampled every 1,000 generations for 7.5x10^6^ (nSSU), 7.5x10^6^ (nLSU) and 5x10^6^ (COX1) generations. Convergence of Markov chains was assessed using Tracer 1.4 (Rambaut A, Drummond AJ (2007); available from http://beast.bio.ed.ac.uk/Tracer) and saved trees from the first 750,000 (nSSU, nLSU), 500,000 (Spirurina) and 300,000 (COX1) generations were discarded as burn-in.

### Statistical parsimony network analysis

Unique sequence types per population of *A. crassus* (defined in Table 3; including gaps as a 'fifth state') were collated as an aligned NEXUS format file (see Additional file 1), and TCS 1.2.1 [70] was used to estimate gene genealogies within populations [71]. The network graphs were output in graph markup language and visualised and annotated with custom perl scripts and 4yEd (version 3.4.2). 

**Table 3:** **Population designations for *Anguillicola crassus *population structure analyses with COX1 **

**Group**	**Prefix**	**Population**	**No. sequences**	**Source**
North-Eastern Europe	ALA	Åland Islands (Finland)	16	[19]
North-Eastern Europe	OER	Kullen, Øresund/Kattegat (Sweden)	30	[19]
North-Eastern Europe	COR	Slapton Ley, Cornwall (Great Britain)	15	[19]
North-Eastern Europe	NEA	Lake Neagh (Great Britain)	31	[19]
North-Eastern Europe	SHA	Lough Dergh, Shannon (Ireland)	30	[19]
North-Eastern Europe	GRA	Rußheimer Altrhein (Germany)	4	*
North-Eastern Europe	GST	Steinfeld (Germany)	1	*
North-Eastern Europe	POL	Sniardwy Lake, Mikolajki (Poland)	5	*
North-Eastern Europe	C	Essen (Germany)	3	**
Brittany	FRE	Bois Joli, Frémur (France)	31	[19]
Brittany	VIL	Brain-sur-Vilaine (France)	30	[19]
South-West Europe	LOI	Angers, Loire (France)	32	[19]
South-West Europe	RHO	Camargue, Rhône (France)	30	[19]
South-West Europe	ORI	Oria (Spain)	30	[19]
South-West Europe	EAV	Albufera de Valencia (Spain)	3	*
South-West Europe	POR	Ribeira das Lampreias (Portugal)	1	*
South-West Europe	TIB	Roma, Tiber (Italy)	30	[19]
South-West Europe	LB	Lake Bracchiano (Italy)	10	**
Turkey	TUR	Asi River, Hatay (Turkey)	4	*
USA	STJ	St. Jones River (New Jersey, USA)	32	[19]
Taiwan	KAO	Tung-Chiang, Kao-Ping River (Taiwan)	46	[19]
Taiwan	TKR	Sinyuan, Kaoping River (Taiwan)	4	*
Taiwan	TCU	Eel culturing pond, Budai, (Taiwan)	5	*
China (Zhuhai)	CGZ	Zhuhai, Guangdong (China)	14	*
China (Guangzhou)	CGG	Guangzhou, Guangdong (China)	2	*
Japan (Mikawa)	MIK	Mikawa Bay (Japan)	29	[19]
Japan (Yamaguchi)	YAM	Yamaguchi, Fushino (Japan)	7	[19]
Japan (Wakayama)	JPN	Natural water system, Wakayama (Japan)	5	*

* This work.

** Geiß, K. and B. Sures (2010). The swim bladder nematode *A. novaezelandiae*. Proceedings of the Joint Meeting of the German Societies of Parasitology and Protozoology, Düsseldorf, Germany.

## Competing interests

The authors declare that they have no competing interests.

## Authors’ contributions

DL performed the molecular work and analysed the data. HT facilitated and coordinated the collection of specimens. HT and EH collected some of the samples. SN provided essential DNA sequences. The manuscript was written by DL and MB. EH, HT and SN edited the manuscript. All authors read and approved the manuscript.

## Supplementary Material

Additional file 1 **Sequence data files and keys thereto as a single compressed file, expandable to a folder containing:Cytochrome oxidase 1 data,** COX1.haplotypes.nex - NEXUS format file of aligned COXI haplotype sequences, COX1.haplotypes.labels.txt - text file describing the assignment of individual COXI sequences to haplotypes, COX1.haplotypes.crassus.nex - NEXUS format file of aligned *A. crassus* COXI haplotype sequences, COX1.haplotypes.crassus.labels.txt - text file describing the assignment of individual *A. crassus* COXI sequences to haplotypes, COX1.nex - NEXUS format file of all aligned COXI haplotype sequences. **Nuclear large subunit ribosomal RNA data,** 28_S.haplotypes.nex - NEXUS format file of aligned nLSU or 28S haplotype sequences, 28_S.nex - NEXUS format file of all aligned nLSU or 28S haplotype sequences. **Nuclear small subunit ribosomal RNA data,** 18_S.haplotypes.nex - NEXUS format file of aligned nSSU or 18S haplotype sequences, 18_S.spirurina_B.nex - NEXUS format file of aligned nSSU or 18S haplotype sequences, 18_S.nex - NEXUS format file of all aligned nSSU or 18S haplotype sequences. Click here for file
